# Supplementary material for: Resolving the full spectrum of human genome variation using Linked-Reads
Source: Genome Res. 2019 Apr;29(4):635–45. doi: 10.1101/gr.234443.118 (PMC6442396; doi:10.1101/gr.234443.118)
Supplement: Supplemental Material [file supp_gr.234443.118_Supplemental_longranger-2.2.zip › longranger-2.2.tar.bz2!/lr2.2-public/lib/stan/cmdstan/stan/doc/api/html/multi__student__t_8hpp.html]

Stan: src/stan/prob/distributions/multivariate/continuous/multi\_student\_t.hpp File Reference


|  |  |
| --- | --- |
|  | Stan  2.5.0  probability, sampling & optimization |


- Main Page
- Related Pages
- Namespaces
- Classes
- Files

- File List
- File Members

All Classes Namespaces Files Functions Variables Typedefs Enumerations Enumerator Friends Macros Pages

- src
- stan
- prob
- distributions
- multivariate
- continuous

Namespaces |
Functions

multi\_student\_t.hpp File Reference

`#include <cstdlib>`  
`#include <boost/math/special_functions/gamma.hpp>`  
`#include <boost/math/special_functions/fpclassify.hpp>`  
`#include <stan/math/matrix.hpp>`  
`#include <stan/math/error_handling.hpp>`  
`#include <stan/math/matrix/multiply.hpp>`  
`#include <stan/math/matrix/dot_product.hpp>`  
`#include <stan/math/matrix/subtract.hpp>`  
`#include <stan/math/matrix_error_handling.hpp>`  
`#include <stan/prob/constants.hpp>`  
`#include <stan/prob/traits.hpp>`  
`#include <stan/prob/distributions/multivariate/continuous/multi_normal.hpp>`  
`#include <stan/prob/distributions/univariate/continuous/inv_gamma.hpp>`  
`#include <stan/math/error_handling/matrix/check_ldlt_factor.hpp>`  
`#include <boost/random/variate_generator.hpp>`

Go to the source code of this file.

|  |  |
| --- | --- |
| Namespaces | |
|  | stan |
|  | Probability, optimization and sampling library. |
|  | |
|  | stan::prob |
|  | Templated probability distributions. |
|  | |

|  |  |
| --- | --- |
| Functions | |
| template<bool propto, typename T\_y , typename T\_dof , typename T\_loc , typename T\_scale > | |
| boost::math::tools::promote\_args  < typename scalar\_type< T\_y >  ::type, T\_dof, typename   scalar\_type< T\_loc >::type,   T\_scale >::type | stan::prob::multi\_student\_t\_log (const T\_y &y, const T\_dof &nu, const T\_loc &mu, const Eigen::Matrix< T\_scale, Eigen::Dynamic, Eigen::Dynamic > &Sigma) |
|  | Return the log of the multivariate Student t distribution at the specified arguments. More... |
|  | |
| template<typename T\_y , typename T\_dof , typename T\_loc , typename T\_scale > | |
| boost::math::tools::promote\_args  < typename scalar\_type< T\_y >  ::type, T\_dof, typename   scalar\_type< T\_loc >::type,   T\_scale >::type | stan::prob::multi\_student\_t\_log (const T\_y &y, const T\_dof &nu, const T\_loc &mu, const Eigen::Matrix< T\_scale, Eigen::Dynamic, Eigen::Dynamic > &Sigma) |
|  | |
| template<class RNG > | |
| Eigen::VectorXd | stan::prob::multi\_student\_t\_rng (const double nu, const Eigen::Matrix< double, Eigen::Dynamic, 1 > &mu, const Eigen::Matrix< double, Eigen::Dynamic, Eigen::Dynamic > &s, RNG &rng) |
|  | |


---

[ Stan Home Page ]

*© 2011–2014,
Stan Development Team.*
